# Tentacle Mesh for Fixation-Free Spigelian Hernia Repair: Mini-Invasive Approach Granting Broad Defect Overlap

**DOI:** 10.3390/jcm12123866

**Published:** 2023-06-06

**Authors:** Giuseppe Amato, Antonino Agrusa, Salvatore Buscemi, Giuseppe Di Buono, Pietro Giorgio Calò, Roberta Vella, Giorgio Romano, Gabriele Barletta, Giovanni Cassata, Luca Cicero, Giorgio Romano

**Affiliations:** 1 Department of Surgical, Oncological and Oral Sciences, University of Palermo, 90127 Palermo, Italy; 2 Department of Surgical Sciences, University of Cagliari, 09124 Cagliari, Italy; 3 Postgraduate School of General Surgery, University of Palermo, 90127 Palermo, Italy; 4 CEMERIT (Centro Meridionale Ricerca e Training), IZSS, 90129 Palermo, Italy

**Keywords:** MIS, advanced surgical techniques, spigelian hernia, ventral hernia, tentacle mesh, preperitoneal sublay, mesh overlap, surgical fixation devices, fixation-free

## Abstract

Background: Compared to other types of abdominal protrusions, Spigelian hernias are not very common. In prosthetic repair of abdominal protrusions, mesh fixation and defect overlap are an open issue, as they are a source of complications. A newly developed tentacle-shaped mesh has been used to ensure a fixation-free repair with a broader defect overlap in the repair of abdominal hernias. This study describes the long-term results of a fixation-free repair of Spigelian hernias carried out with a tentacle mesh. Methods: A proprietary mesh composed of a central body with integrated radiating arms was used for repairing Spigelian hernias in 54 patients. The implant was positioned in preperitoneal sublay, and the straps were delivered across the abdominal musculature with a needle passer, and then, after fascia closure, cut short in the subcutaneous layer. Results: The friction of the straps passing through the abdominal wall served to hold the mesh in place, guaranteeing a wide overlap over the defect without fixation. In a long-term follow-up of 6 to 84 months (mean 64 months), a very low rate of complications occurred, but no recurrence was reported. Conclusions: The tentacle strap system of the prosthesis allowed for an easy, fast and safe fixation-free placement granting a wide overlap, avoiding intraoperative complications. Greatly reduced pain and a negligible amount of postoperative complications characterized the postoperative outcome.

## 1. Introduction

**Figure 1:**
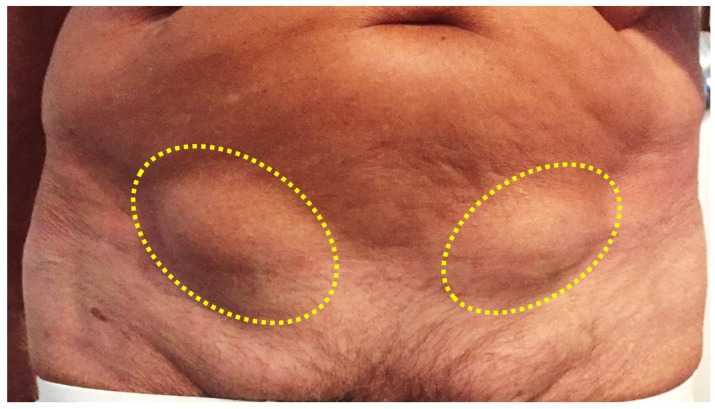
Bilateral Spigelian hernia in a male patient (yellow dotted ovals).

Spigelian hernia accounts for about 2% of all hernias and, therefore, is relatively infrequent [1]. In 1645, Adriaan van der Spiegel, a Flemish anatomist, first described a defect in the semilunar line (linea Spigeli) (Figure 1). Later, in 1764, Josef Klinkosch defined the Spigelian hernia as a defect in the semilunar line; this region is referred to as “the Spigelian hernia belt” [2]. The Spigelian aponeurosis is located between the semilunar line and the lateral edge of the rectus muscle [3]. Most Spigelian hernias occur on the right side and between the fourth and seventh decades of life, affecting women more than men [4]. In a large case series, a female/male ratio of 1.7/1 was reported [5]. It can be congenital or acquired, and a wide range of pathologies may act as predisposing factors [6,7]. Although multiple conditions predispose to developing Spigelian hernias, recent reports have found that at least 50% of all patients with Spigelian hernias had previously undergone abdominal surgery including both open and laparoscopic operations [8,9]. There are two types of Spigelian hernias: protrusions located above the inferior epigastric vessels and those located caudally to the latter [10]. In most cases, the hernial sac contains the omentum, but in some cases it may also contain a segment of small intestine or colon that may cause intestinal obstruction. Several cases of congenital Spigelian hernia with undescended testis have also been reported [11,12]. Preoperative diagnosis is challenging, as the sac is usually located between the muscle layers of the abdominal wall, and therefore abdominal computed tomography (CT) or ultrasound (US) is usually the first choice for confirming the diagnosis. The defects of Spigelian hernias are usually small, and therefore the risk of strangulation is high. [13] As the incarceration rate of Spigelian hernia is very high, ranging from 17% to 33%, prompt surgery should be performed [14,15].

The surgical approaches to Spigelian hernias vary from a traditional open hernia repair using primary sutures or mesh to laparoscopic techniques, which in the past 20 years have become much more common [5,16].

At present, however, prosthetic repair of Spigelian hernia is unanimously considered the gold standard, for both open and laparoscopic approaches. In prosthetic repair, implant fixation is necessary to avoid migration. Nevertheless, fixation of the mesh in the myotendinous arrangement of the abdominal wall implicates the possible occurrence of post-operative adverse events such as bleeding or hematomas. In addition, sutures or tacks used to fasten the implants can tear the tissue. As a consequence, meshes become unfastened, and migration can occur, thus facilitating recurrences [17]. In prosthetic hernia repair, mesh overlap upon the defect is crucial. Starting from a few weeks after placement, the synthetic material begins to shrink, and this may lead to defect de-coverage that is a prelude of a recurrence. Moreover, guaranteeing a wide defect overlap is essential in the case of Spigelian hernia repair. In light of the above, a newly designed implant intended to be placed in preperitoneal sublay was used to manage Spigelian hernia with an open approach. This newly developed implant is structured with a central oval body having eight bands incorporated at its edge. These straps are delivered with a specific needle passer from the preperitoneal space across the musculature and fascia to the subcutaneous layer. This approach allows for an easy, rapid, fixation-free deployment of the implant ensuring a broad overlap over the defect. The friction exerted by the tentacle straps crossing the abdominal musculature holds the mesh in place without need for suture stitches or tack fixation. The force exerted by the straps tunneled through the tissues is sufficiently strong to avoid mesh dislocation. The effectiveness of this principle has already been experimentally tested on a large animal model, and the tentacle mesh is currently employed for surgical treatment of incisional, umbilical and ventral hernia repair [18,19,20,21,22]. The aim of this study is to demonstrate the effectiveness of a fixation-free repair of Spigelian hernias with the described tentacle-shaped mesh.

## 2. Material and Methods

This research was performed in accordance with the Declaration of Helsinki for experiments involving humans. Institutional Ethics Committee ethically approved this study. Informed written consent was signed by all participants in this study. A cohort of 54 patients diagnosed with Spigelian hernia who consecutively underwent open surgical repair with the tentacle-shaped implant (Freedom Octomesh VHR XS produced by Insightra Medical Inc., Clarksville, TN, USA) forms the body of this study.

### 2.1. The Device

**Figure 2:**
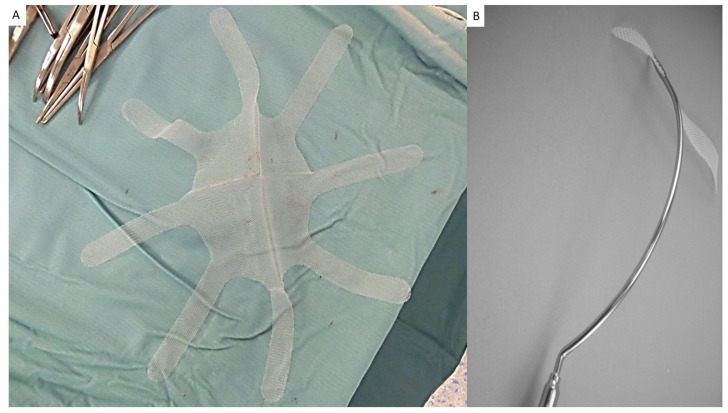
(**A**) Tentacle-shaped implant Freedom Octomesh VHR Type XS with a central oval body and 8 straps at the edge of the prosthesis. (**B**) The needle passer used for the delivery of straps from preperitoneal space across abdominal wall structures to subcutaneous layer.

The tentacle-shaped prosthesis specifically used for the mini-invasive procedure consists of a proprietary oval flat mesh measuring 12 × 15 cm, with eight straps, 15 cm in length and 2 cm in width, placed at regular distances at the edge (Figure 2A). The mesh, manufactured with lightweight, large-pore polypropylene fabric, with a density of 70 g/m^2^ and pores > 10 µm, is intended to be positioned in preperitoneal sublay through the hernia defect by means of a specially designed needle passer with incorporated eye (Figure 2B).

### 2.2. Procedural Steps

**Figure 3:**
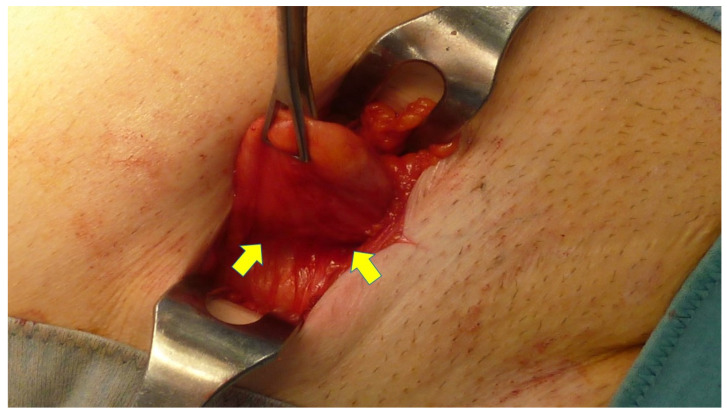
Right-sided partly obstructed Spigelian hernia protrusion emerges from the fascial defect in arcuate line. Stricture at the basis of the protrusion (yellow arrows) causes the obstruction of hernia content within the sac.

**Figure 4:**
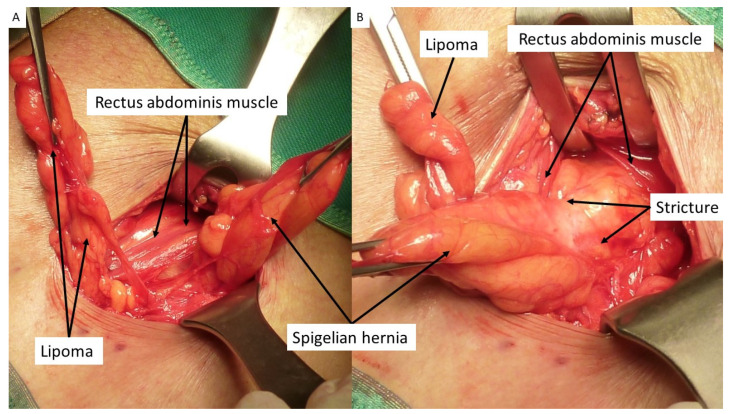
(**A**) After opening the fascia, an obstructed Spigelian hernia with tightened basis is detected in the lateral margin of rectus abdominis muscle. A lipoma is also visible at the opposite margin. (**B**) After lateral dislodgement of hernia sac and displacing the rectus muscle medially, the thickened stricture of the sac constraining the hernia opening is clearly visible.

**Figure 5:**
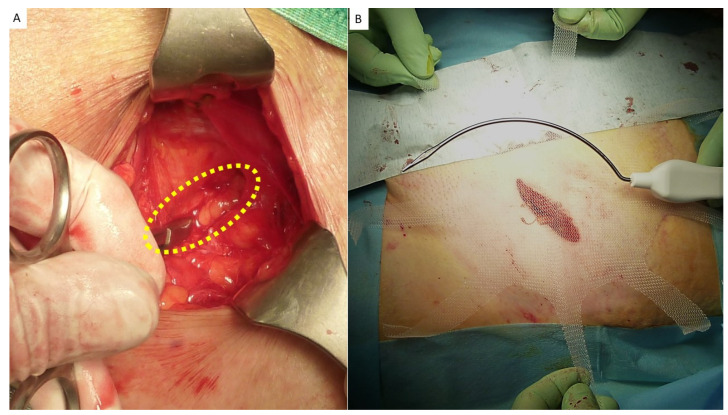
(**A**) Protrusion is returned to abdominal cavity by means of forceps. Hernia defect is clearly detectable (yellow dotted oval). (**B**) Tentacle mesh is brought close to operation site prior to delivery. Note: wide surface of the implant that will be placed in preperitoneal sublay.

**Figure 6:**
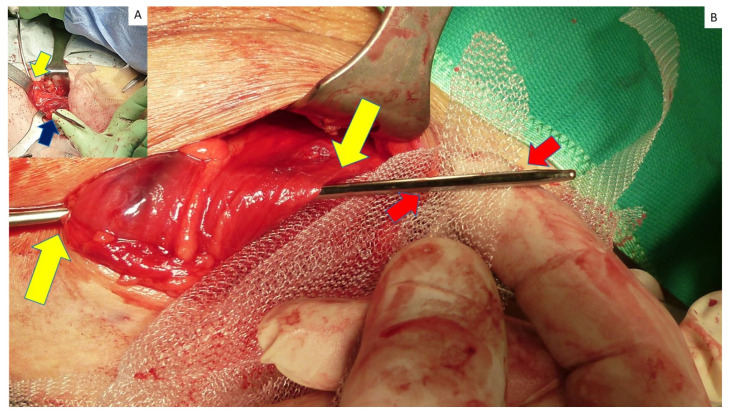
(**A**) After blunt dissection of the preperitoneal space to achieve a wide space for mesh placement, the tip of the needle passer pierces fascia penetrating though the muscle layer to the preperitoneal space (yellow arrow). Maneuver is facilitated by introduction of forefinger tip in the preperitoneal interstitium between muscle and peritoneum. Fingertip guides the advancement of the needle outside the lateral margin of rectus muscle (blue arrow), avoiding a tear of the peritoneal sheath. (**B**) First strap of the tentacle mesh is inserted into the eye of the needle passer (red arrows) passing through the preperitoneal space, crossing muscular layer distant from the defect border. Yellow arrows indicate the overlap ensured by this procedural step.

**Figure 7:**
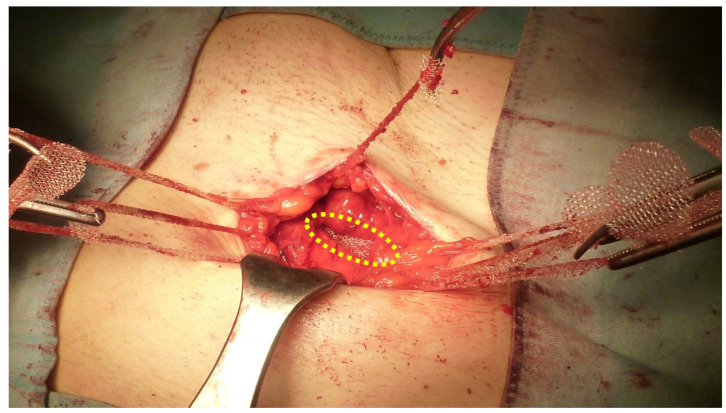
All eight straps are delivered across the muscle layers. Body of the mesh lies in the preperitoneal space posteriorly of abdominal wall (yellow dotted oval). Pulling the straps high allows the mesh to be automatically deployed flat over the peritoneal sheath.

**Figure 8:**
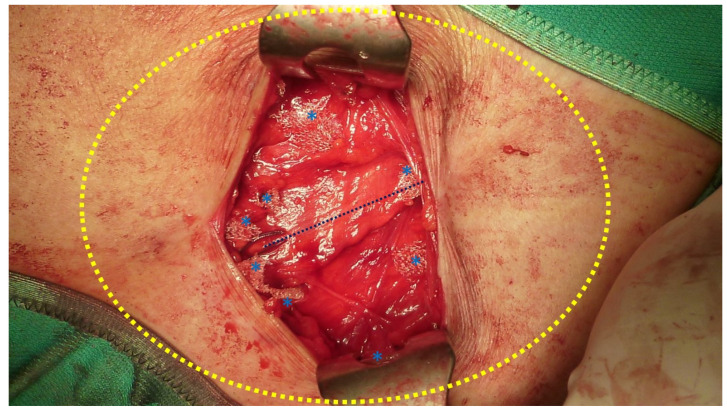
After positioning the tentacle mesh preperitoneally, fascia is closed with continuous resorbable suture. Here, each strap (blue *) is cut short leaving a stump of ca. 2 cm in the subcutaneous space. Yellow oval indicates the surface of the preperitoneal space occupied by the tentacle mesh ensuring a wide overlap on the already sutured defect line (dotted blue line).

In addition to clinical examination of the enrolled patients, a US and/or CT scan was carried out. An antibiotic prophylaxis with 2 g cephalosporin IV was administered preoperatively. To carry out the procedure, a skin incision is made in correspondence to the arcuate line where the hernia protrudes. After incising the subcutaneous fat, the hernia sac is dissected until the defect in the fascial layer is clearly visible (Figure 3 and Figure 4A,B). The fascia is then opened as wide as necessary to allow the protrusion to be returned to the abdominal cavity (Figure 5A). If needed, the redundant part of the peritoneal sac can be resected after ligation at the basis. Then, using a mounted pad, a blunt dissection of the peritoneal sheath from the posterior abdominal wall is carried out. A preperitoneal space as distant as possible from the hernia opening can thus be created, wide enough for the placement of the prosthetic body. At this stage, the procedure of positioning the tentacle mesh can be initiated by delivering all 8 straps across the abdominal wall layers to the subcutaneous layer with the aid of a specific needle passer (Figure 5B). The spike of this proprietary curved elongated device is introduced across the abdominal wall to the dissected preperitoneal space. To avoid injuries of the abdominal content, the interposition of the forefinger guides the advancement of the needle spike in a blind fashion until the margin of the defect is reached (Figure 6A). The straps are then inserted in the needle eye and delivered laterally of the defect at a distance far enough to ensure a wide overlap (Figure 6B). Having passed through the abdominal wall, all eight straps are pulled high, thus allowing for a flat deployment of the mesh body in the preperitoneal layer (Figure 7). The friction exerted by the eight straps, fixation-free fastens the mesh body to the abdominal wall. Once the straps are positioned, the hernia defect is closed over the implant with resorbable sutures. After closure of the fascia, the straps are cut short in the subcutaneous layer leaving a stump of ca. 2 cm (Figure 8). The incision can then be closed with intradermal skin sutures. A protocol based analgesic medication with paracetamol is administered until the 3rd day post operation.

### 2.3. Follow-Up Protocol

Postoperative follow-ups were planned at 1, 3, 6, 12 months, and each subsequent year. In addition to physical examination, the follow-up also included the use of the visual analogue scoring system (VAS) to assess pain and US control to examine implant deployment and tentacle position.

## 3. Results

**Figure 9:**
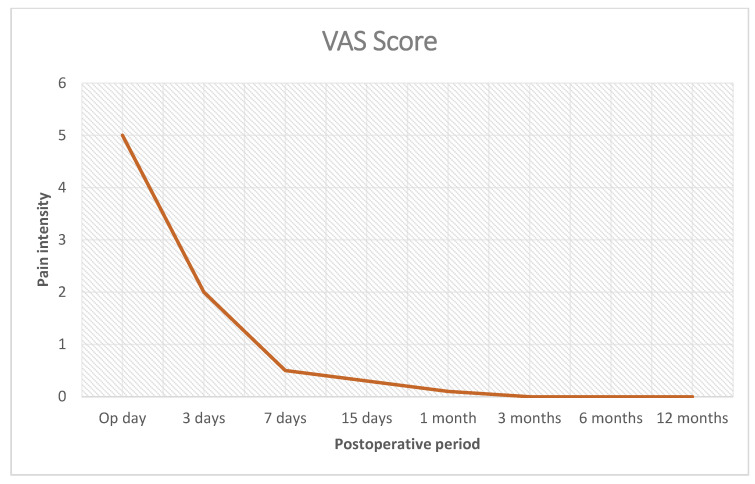
Postoperative pain intensity (visual analogue scale).

The procedure was performed in 54 patients (24 men and 30 women with a mean age of 64 years (range 53 to 76) and a median BMI of 27.40 (range 24–31). Forty-eight repaired Spigelian hernias were primary protrusions (seven incarcerated), while the additional six were recurrent protrusions (among these two multi-recurrent and one incarcerated). Mean hernia defect width was 2.5 cm (range 1.5–3 cm). Patient demographics and hernia types are detailed in Table 1. Concerning the surgical procedure, forty-five patients were operated in assisted local anesthesia (with sedation when needed) and nine in general anesthesia. In all 54 patients, the tentacle-shaped implant was placed in preperitoneal sublay. All implants were positioned in a fixation-free fashion with a mean mesh overlap of ca. 6 cm assessed from the hernia border to the lateral edge of the prosthesis. Due to the variability of hernias repaired, duration of the mesh placement was determined starting from the return of the protrusion into the abdominal cavity and ending with the placement of the last tentacle. Overall, the time needed for these steps, including strap delivery, mesh placement and strap positioning, lasted, on average, 5 min (range from 4 to 7 min). No fixation of the implant was required to hold the body of the tentacle mesh in place. All surgeries, except those with incarcerated hernias, were carried out in day surgery with patients discharged within 24 h of the procedure (Table 2). In the postoperative period, we observed four (7.41%) seromas that were successfully managed conservatively and resolved within 15 days. No wound infection, hematoma, chronic pain or recurrence were reported in the follow-up, ranging from 6 to 84 months (Table 3). During the follow-up, patients were asked about numbness and/or pain, limitations of abdominal-wall movements and overall satisfaction. Above all, patient satisfaction was high and, with regard to postoperative pain assessed using the Visual Analog Scale (VAS), our findings were encouraging. In detail, three days after the operation patients reported an average pain score of approximately three VAS points. By day 7, the VAS score had decreased significantly, indicating minimal pain. Remarkably, from 2 weeks postoperative and beyond, patients reported no further pain even during loading movements, as shown in Figure 9. These results suggest that the surgical procedure was successful in managing postoperative pain, with patients experiencing a rapid and sustained recovery. In addition, even in the long-term postoperative period, patients did not report any mesh-related discomfort, especially in the subcutaneous layer due to position of the strap stumps. The proprietary design of the tentacle mesh postoperatively allowed for an easy evaluation of mesh position through US scans during the follow-up thanks to a clear detection of all tentacles placed at regular distances around the abdominal wall. In effect, incorrect placement of the tentacles could reveal an eventual implant dislocation. However, in the described patient cohort, no mesh dislocation was documented.

## 4. Discussion

Spigelian hernia is commonly perceived as a rare disease that deserves no particular attention. Despite this, many patients suffering with Spigelian protrusions experience adverse events that could be avoided if properly diagnosed and managed. The latter also implies improving the operative strategy by using dedicated prosthetic devices to avoid intra and postoperative mishaps, thus granting patients a smooth recovery. Even though a small Spigelian hernia is often managed with direct suture of the defect, today, prosthetic repair represents the preferred choice for the treatment of this kind of abdominal wall protrusion. Open or laparoscopic techniques with the deployment of a flat mesh are the most frequently used methods. Some studies involving large cohorts of patients indicate a recurrence rate of ca. 4% after prosthetic Spigelian hernia repair [1]. However, several factors may influence the development of a recurrence in this type of repair: BMI, BPCO, intestinal disorders with constipation, size of the defect and, in the case of prosthetic repair, implant migration and insufficient mesh overlap. The overlap of the mesh covering the defect is an important issue that is increasingly pointed out in the literature. Currently, a minimum of 5 cm prosthetic overlap is recommended to repair an abdominal wall defect [23,24]. This seems to be a major issue that can facilitate a recurrence, especially in patients with large defects. Another significant aspect is mesh fixation: in open repair, placing suture stitches at the distant boundary of the mesh in dark narrow spaces is often extremely challenging. Intraoperative mesh fastening upon the abdominal musculature, with sutures in open repairs or tacks in laparoscopy, frequently leads to complications such as tissue tear and bleeding with subsequent waste of time. Postoperative detachment of sutures or tacks with tissue tear may also occur; this facilitates the development of hematoma and/or mesh dislodgment [21]. In this regard, it should be also taken into account that mesh positioning in the abdominal wall layers plays a significant role. It is well acknowledged in the literature that the deeper the mesh is placed, the better the results obtained to avoid recurrences are [19,20,21,22]. For this reason, the implant deployment in preperitoneal sublay appears more suitable to prevent postoperative reappraisal of the protrusion. The tentacle-shaped implant described herewith has just been developed to overcome all these issues in the surgical treatment of ventral and incisional hernias. It has been tested with excellent results in an experimental trial on porcine animal model. This experimental attempt served to finalize the steps of the procedure allowing for the preclinical validation of the surgical technique [18]. The procedural steps outlined in section Material and Methods involve the separation of the preperitoneal layer between the posterior abdominal wall and peritoneal sheath to ensure a wide space for the deployment of the prosthesis. Once the dissection of the preperitoneal planes has been carried out, delivery of the tentacle mesh using the proprietary passer is easy, rapid and safe. This device facilitates an uncomplicated placement of the tentacle straps carrying these across the abdominal wall from the preperitoneal space to the subcutaneous layer. Sightly interposing the fingertip between the posterior surface of the abdominal wall and the dissected peritoneum makes a safe penetration and the guidance of the needle spike to load the strap into the needle eye possible; thus, the deployment of the implant edges distant enough from the hernia border can be ensured. In practice, the friction exerted by the tentacle straps crossing the abdominal wall layers laterally from the defect is sufficient to permanently hold the prosthesis in place guaranteeing a wide overlap. Briefly, the 3 mm thick needle passer funneled through the abdominal wall tissue forces the 2 cm wide strap to roll along the axis. This principle of physics, already recognized in the literature, appears to be an appropriate way to ensure a fixation-free, but firm placement, of the polypropylene bands used for the treatment of female genital prolapse [25,26,27,28,29]. Therefore, the friction of the rolled strap surface in the narrow passageway allows for a stable placement amid the tissues crossed. This results in a completely fixation-free prosthetic deployment that, unlike the conventional point-to-point sutures or tacks applied to fix the mesh edges, can be safely and comfortably performed. Furthermore, excluding the challenging steps of point fixation effectively simplifies and speeds up the intervention, allowing for a rapid strap delivery and implant deployment. The greatly reduced postoperative number of complications, only four seromas for a total complications rate of 7.1%, achieved using the Spigelian hernia repair technique described, with the tentacle mesh, appears to be a consequence of reduced surgical trauma and simplified procedure. The US carried out during the follow-up allowed for a clear detection of the tentacle strap stumps, which could be helpful in terms of recurrence prediction that, however, to date has not occurred. The US detection of the tentacle strap stumps also guaranteed the effective assessment of the mesh overlap from the hernia border, which in all patients ranged from 5 to 7 cm, thus, an excellent mesh overlap in this kind of procedure. The wide fixation-free coverage of the preperitoneal surface achieved with the tentacle mesh likely explains why no recurrences occurred in the described cohort. The much reduced postoperative pain, which completely faded within a few days, and the consequent quick return to daily incumbencies clearly appear to be a consequence of the diminished trauma following the fixation-free procedure.

However, if a comparison is made between the features of the described open Spigelian hernia repair with the tentacle mesh and those of the laparoscopic approaches, some significant considerations emerge. The tentacle mesh repair is fully fixation-free and therefore pain sparing. On the contrary, especially in laparoscopic repair, the mandatory use of fixation tools (tacks) that sharply pierce the musculature negatively impacts on abdominal wall movements. This is unanimously considered the cause of increased postoperative pain and complications such as tissue tear, bleeding and hematoma [30,31]. In general, if detachment of the fastening tools occurs, an unfastened mesh may dislodge, increasing the risk of recurrence [32]. Moreover, if a tack drops into the abdominal cavity, also bowel perforation may occur [33,34,35]. A further advantage of the open surgical approach with the tentacle mesh is the true mini-invasive nature of the repair that in nearly all cases can be carried out quickly and safely in assisted local anesthesia through a small skin incision. This allows for an uncomplicated patient discharge within 24 h. These factors play an important role in increasing patient satisfaction and containing the overall costs of the procedure, which in laparoscopic repair are evidently higher.

## 5. Conclusions

Spigelian hernia repair with the described tentacle mesh demonstrated excellent outcomes in the described cohort of patients. The benefits derived from the use of the tentacle mesh in Spigelian hernia repair seem to be evident and can be summarized as follows:

(a) Simplified mini-invasive procedure with a fully fixation-free implant deployment;
(b) Wide overlap of the hernia defect ensuring a broad coverage of the posterior abdominal wall;
(c) Short duration of the surgical procedure with the absence of intraoperative complications;
(d) Minimal pain allowing for an outpatient system procedure and quick return to daily activities;
(e) Negligible rate of postoperative complications; no chronic pain;
(f) Easy postoperative US detection of the implant elements in light of recurrence prediction.

It should also be stressed that this study contains limitations. One limitation suggests the need for a comparative analysis to more accurately evaluate the outcomes of the described technique. In order to further enhance the reliability and generalizability of our findings, it is important to compare results of the present study with those obtained using alternative approaches. Therefore, future research should aim to conduct a randomized trial comparing the effectiveness of the Spigelian herniorrhaphy using the tentacle-shaped mesh with the conventional mesh repair, both open or laparoscopic. This will provide a more comprehensive understanding of the potential benefits and limitations of the approach described herein and may inform the development of more effective strategies in the future. Another limitation concerns the number of patients in the study cohort, which despite being statistically significant, at first glance, might be perceived as not representative enough. This was mainly due to two associated factors: the relative infrequency of this specific protrusion disease and the concomitant COVID-19 pandemic that in the past three years has significantly impacted clinical care. However, this supposed limitation is mitigated by similar, positive results of previous studies dealing with ventral and incisional hernia repair carried out with the same tentacle-shaped prosthesis and treatment concept [18,19,20,21].

## Figures and Tables

**Table 1:** Patient demographics and hernia types.

Patient Characteristics	Hernia Types
Number of patients: 54 (24 male–30 female)	Primary Spigelian hernia: 48 (7 incarcerated)
Mean BMI: 27.40 (24–31)	Recurrent Spigelian hernia: 6 (2 multi-recurrent–1 incarcerated)
Mean age: 64 years (53–76)	Mean hernia defect size: 2.5 cm (1.5–3 cm)

**Table 2:** Details of the surgical procedure.

Procedure Details	
Anesthesia	Local: 45 General: 9
Mean mesh overlap	6 cm (5–7 cm)
Time needed for mesh placement and strap positioning	Mean: 6 min (range 4–8 min)
Hospital stay	
Uncomplicated patients: 46	1 day
Patients with incarcerated hernias: 8	2 days

**Table 3:** Postoperative complications.

Postoperative Complications Mean Follow-Up Length: 64 Months (6–84 Months)	
Wound infection	0 (0%)
Seroma	4
Recurrence	0 (0%)
Total complication rate	7.41%

## Data Availability

All data supporting the reported results are available upon request from the corresponding author.
